# Multimodality imaging of renal lymphoma and its mimics

**DOI:** 10.1186/s13244-022-01260-1

**Published:** 2022-08-13

**Authors:** Trinh Nguyen, Akshya Gupta, Shweta Bhatt

**Affiliations:** 1 MD Anderson Cancer Center, 1515 Holcombe Blvd, Houston, TX 77030 USA; 2 University of Rochester Medical Center, Rochester, NY USA; 3 Mayo Clinic, Jacksonville, FL USA

**Keywords:** Renal lymphoma, Ultrasound, Computed tomography, Positron emission tomography, Magnetic resonance imaging

## Abstract

Lymphomatous involvement of the genitourinary system, particularly the kidneys, is commonly detected on autopsies; yet on conventional diagnostic imaging renal lymphoma is significantly underestimated and underreported, in part due to its variable imaging appearance and overlapping features with other conditions. We present a spectrum of typical and atypical appearances of renal lymphoma using multimodality imaging, while reviewing the roles of imaging in the detection, diagnosis, staging, and surveillance of patients with lymphoma. We also illustrate a breadth of benign and malignant entities with similar imaging features confounding the diagnosis of renal lymphoma, emphasizing the role of percutaneous image-guided biopsy. Understanding the spectrum of appearances of renal lymphoma and recognizing the overlapping entities will help radiologists improve diagnostic confidence and accuracy.

## Key points

- The genitourinary system is commonly affected by extranodal spread of lymphoma, with only the reticuloendothelial and hematopoietic systems affected more frequently.
- Six major patterns of renal lymphoma have been described: multiple lesions, solitary lesion, direct extension from retroperitoneal adenopathy, perinephric disease, nephromegaly, and renal sinus involvement.
- Recognizing the typical manifestations of renal lymphoma and differentiating it from common benign and malignant mimics can impact management and obviate unnecessary surgery.

## Background

The genitourinary system is commonly affected by extranodal spread of lymphoma, of which the kidneys are the most commonly involved organ [1]. Primary renal lymphoma is rare, accounting for less than 1% of cases of extranodal lymphoma, and is defined as exclusive involvement of the kidneys without systemic disease [2]. Secondary lymphoma is much more common and frequently found at autopsy, in up to 38% of cases, from the direct spread of retroperitoneal adenopathy or hematogenous spread from systemic disease [3, 4]. Despite the relatively high prevalence of renal lymphoma, imaging studies detect renal abnormalities in only 3–8% of patients undergoing routine staging of disease. [5, 6]

Renal lymphoma has been described in both Hodgkin and non-Hodgkin lymphoma, with non-Hodgkin lymphoma, being far more common [7, 8]. In most cases, renal lymphoma is clinically silent, and radiologic detection seldom influences staging and treatment [4]. The imaging findings can be nonspecific, and there are overlapping features with other benign and malignant conditions which can create a diagnostic dilemma. Six major patterns of renal lymphoma have been described: multiple lesions, solitary lesion, direct extension from retroperitoneal adenopathy, perinephric disease, nephromegaly, and renal sinus involvement [4].

## Main text

### Clinical manifestations

Clinically, patients are relatively asymptomatic however can present with flank pain, hematuria, night sweats, and fever. Acute renal failure rarely manifests as the initial finding in cases of diffuse involvement. And while renal lymphoma can respond to treatment similar to lymphoma elsewhere in the body, recurrent renal lymphoma carries a poorer prognosis [9]. Furthermore, patients who present with acute renal failure in the setting of lymphocytic infiltration of the renal parenchyma can improve their renal function with the treatment of the underlying lymphoma, but typically not back to baseline [10].

### Role of multimodality imaging

CT is the imaging modality of choice for the initial evaluation of patients with suspected lymphoma. The advantages of CT include high sensitivity for detection of renal lesions, extrarenal tumor extension, and involvement of other organs. Intravenous contrast is essential for the detection of subtle lesions; imaging in the late arterial phase is helpful in evaluating the vasculature and differentiating lymphoma from hypervascular primary renal tumors. The nephrographic phase is essential in detecting small lesions. Lymphomatous deposits tend to enhance less than the renal cortex and appear homogenous.

MRI is useful in demonstrating renal and perirenal disease, however, the role of MRI in evaluating renal lymphoma is less clearly defined in the literature. Renal lymphomatous tumors appear as T1 hypointense and T2 iso- or hypointense relative to the renal cortex. On post-contrast MRI, renal lymphoma enhances less than the renal parenchyma, with some lesions demonstrating progressive enhancement on delayed imaging [4].

Ultrasound is often the first imaging exam in patients presenting with renal insufficiency or flank pain. However, ultrasound is inferior to CT and MRI in detecting the presence of disease, number of lesions, and extrarenal disease. Lesions typically appear homogenous and hypoechoic. Color Doppler imaging may demonstrate little internal vascularity within the lesion, which tends to displace vessels rather than invade them [11]. The presence of vascular thrombus in the renal vein or inferior vena cava is atypical for lymphoma and alternative diagnoses should be considered. Contrast-enhanced ultrasound (CEUS) is useful in characterizing lesions and can differentiate between solid tumors, pseudo-lesions, and complex cysts [12]. However, differentiating renal lymphoma from other malignant renal masses is typically not feasible [13].

PET/CT is currently the gold standard for the staging of lymphoma and the detection of recurrent disease [14]. Its advantage in detecting the metabolic activity of tumors makes it more sensitive and specific than conventional anatomic imaging [15].

### Multiple lesions

The most common imaging finding in renal lymphoma, occurring in 50–60% of cases, is multiple solid parenchymal masses [16]. These are more commonly bilateral although multiple unilateral lesions can also occur [17].

**Fig. 1:**
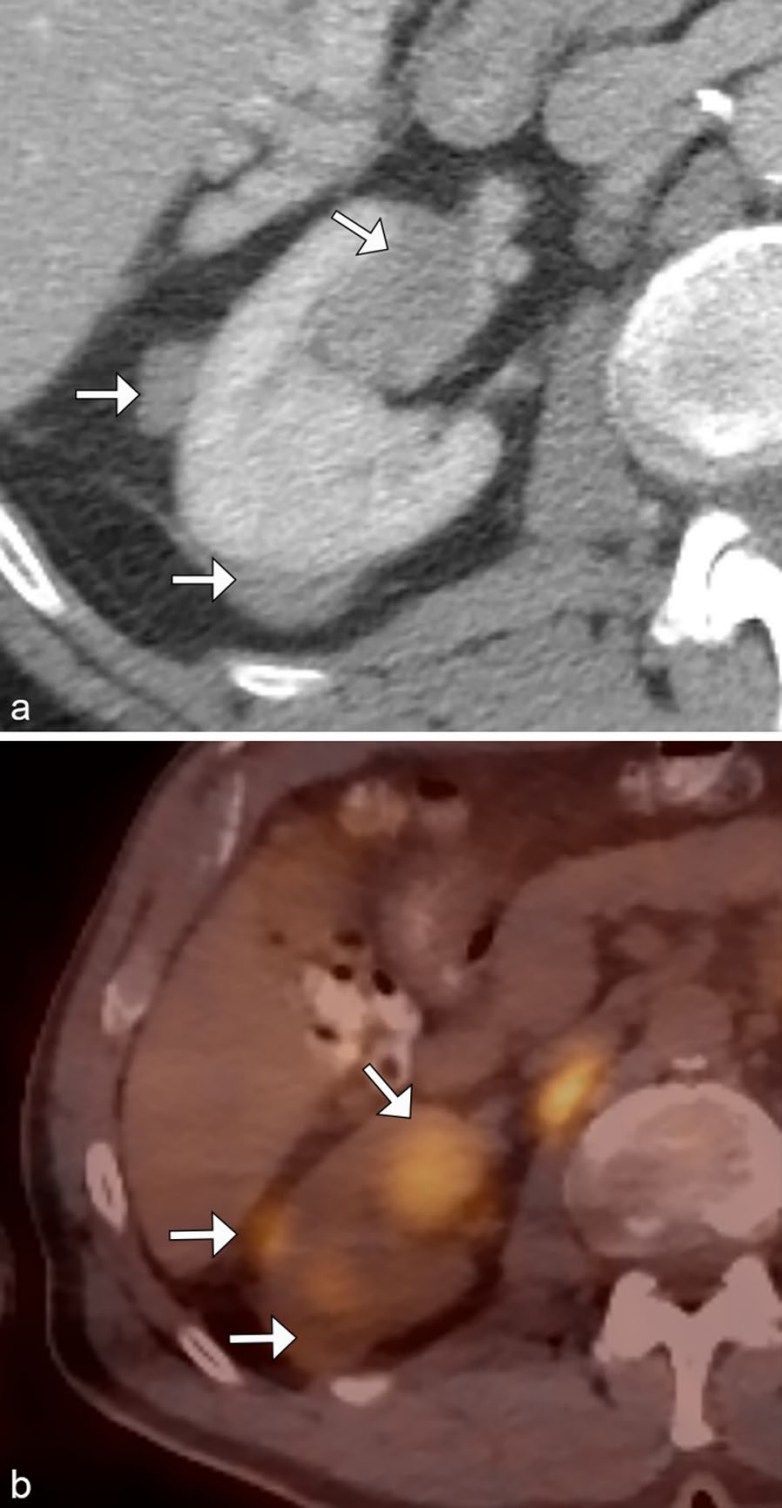
*Multiple renal masses of renal lymphoma*. Axial contrast CT (**a**) demonstrates small, hypoenhancing masses (arrows) in the right kidney. Axial fused PET/CT image (**b**) demonstrates F18-FDG avidity of these masses (arrows) consistent with lymphoma in this patient with known diffuse large b-cell lymphoma

On unenhanced CT, lesions typically have higher attenuation than the surrounding renal parenchyma. Lesions tend to be homogeneous in appearance. (Fig. 1).

The primary differential diagnosis for multiple renal masses includes metastatic disease, commonly from lung, breast, gastric cancer, and melanoma [18]. Multiple synchronous renal cell carcinomas (RCCs), particularly papillary and chromophobe subtypes, can mimic multifocal renal lymphoma. Image-guided biopsy may be needed to differentiate lymphoma from RCC, as the management for lymphoma consists of chemotherapy, not nephrectomy.

Benign differential diagnoses to consider in the setting of multiple renal masses include pyelonephritis, abscesses, renal infarcts, IGG-4 related renal disease, and extramedullary hematopoiesis. The presence of perirenal fascial thickening and infiltration of perinephric fat is nonspecific and has been observed in both inflammatory processes and lymphoma.

### Solitary lesion

**Fig. 2:**
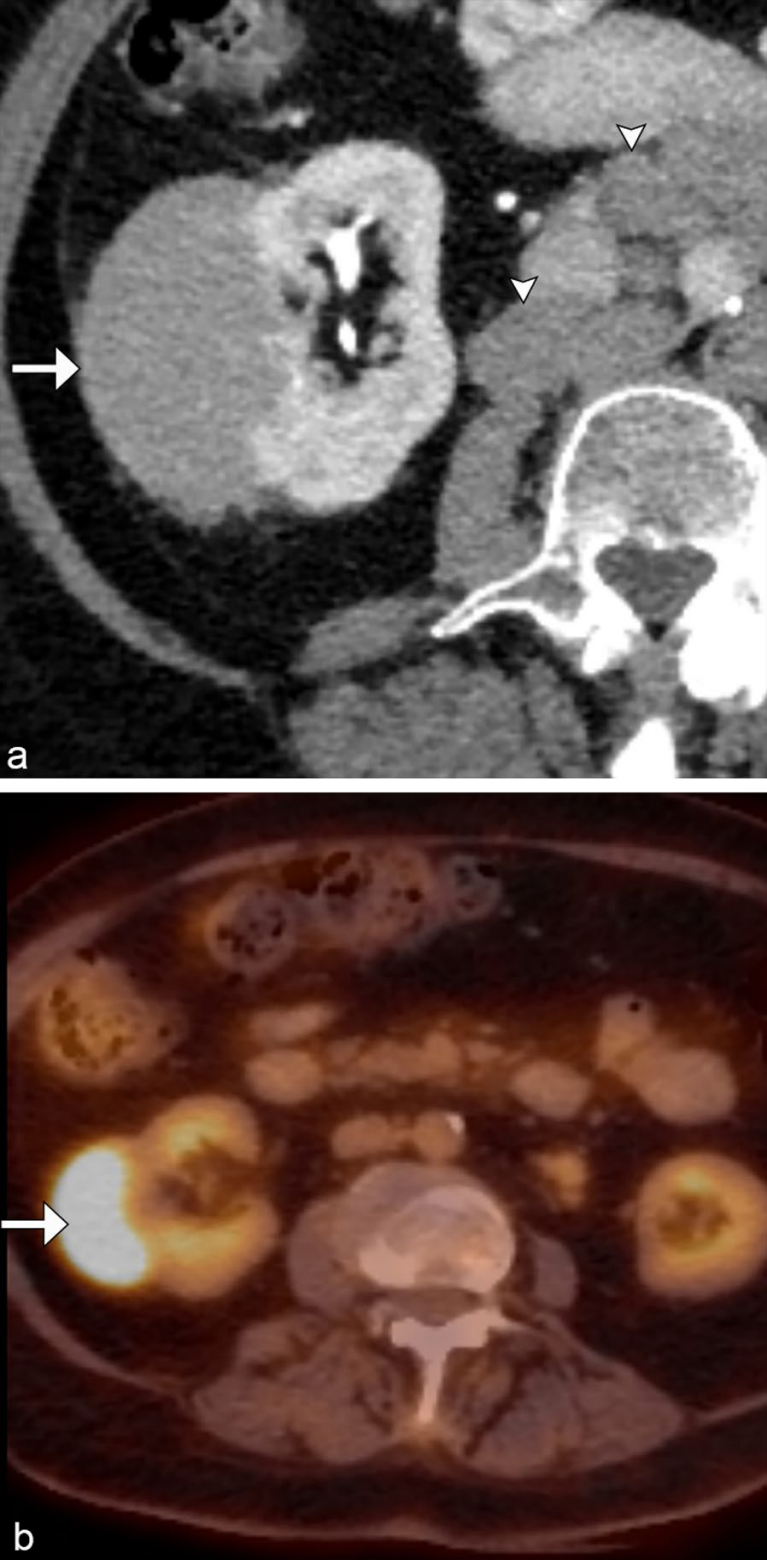
*Solitary mass of renal lymphoma.* Axial contrast CT (**a**) demonstrates a solitary mass (arrow) in the right kidney with avid radiotracer uptake on F18-FDG PET/CT (**b**), as well as retroperitoneal adenopathy (arrowhead). This is consistent with solitary renal lymphomatous mass in this patient with marginal zone lymphoma. The marked FDG avidity differentiates it from typical renal cell carcinoma which can be mildly FDG-avid

**Fig. 3:**
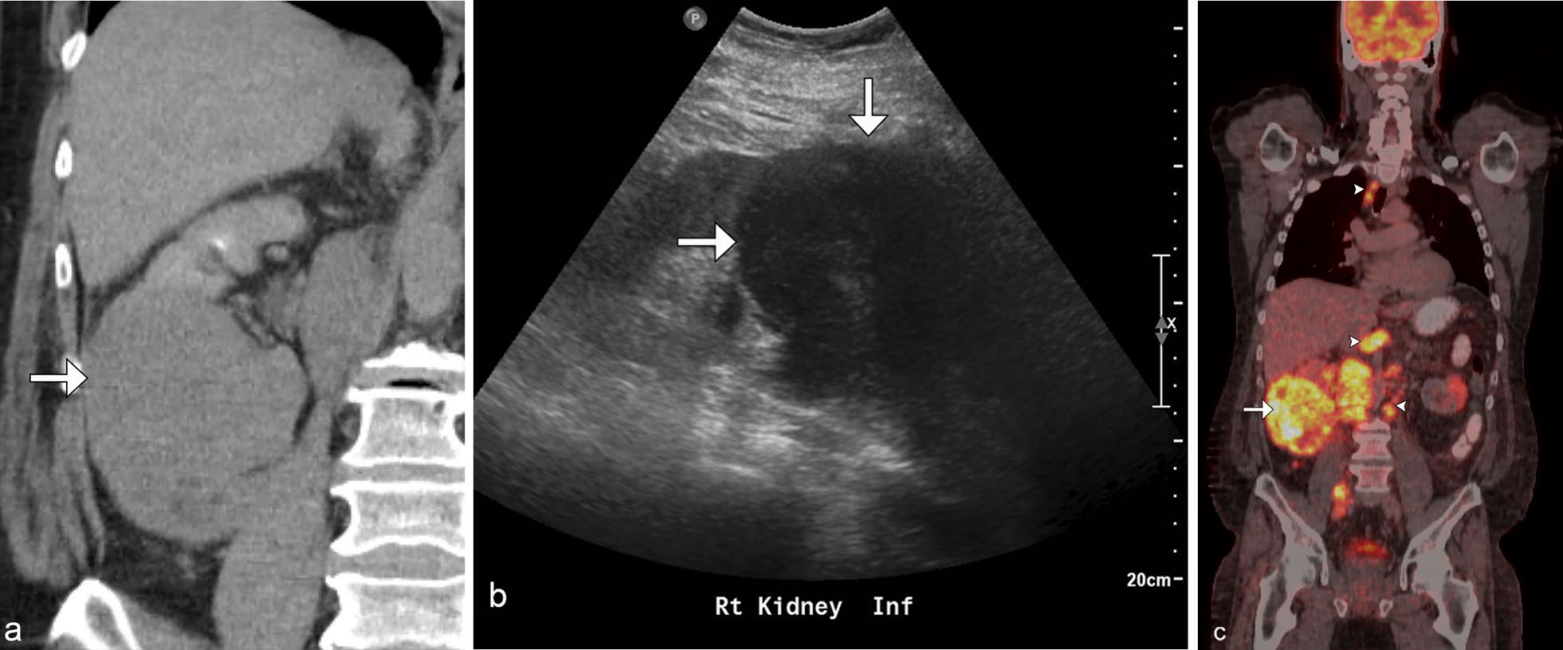
*Solitary mass of renal lymphoma.* Coronal CT delayed post contrast image (**a**) demonstrates a hypoenhancing right lower pole renal mass with hypoechoic appearance on ultrasound (**b**) and marked F18-FDG avidity on PET/CT (**c**) (arrows). Additional FDG avid supradiaphragmatic and extensive infradiaphragmatic lymphadenopathy is helpful in confirming the diagnosis of lymphoma (arrowheads)

**Fig. 4:**
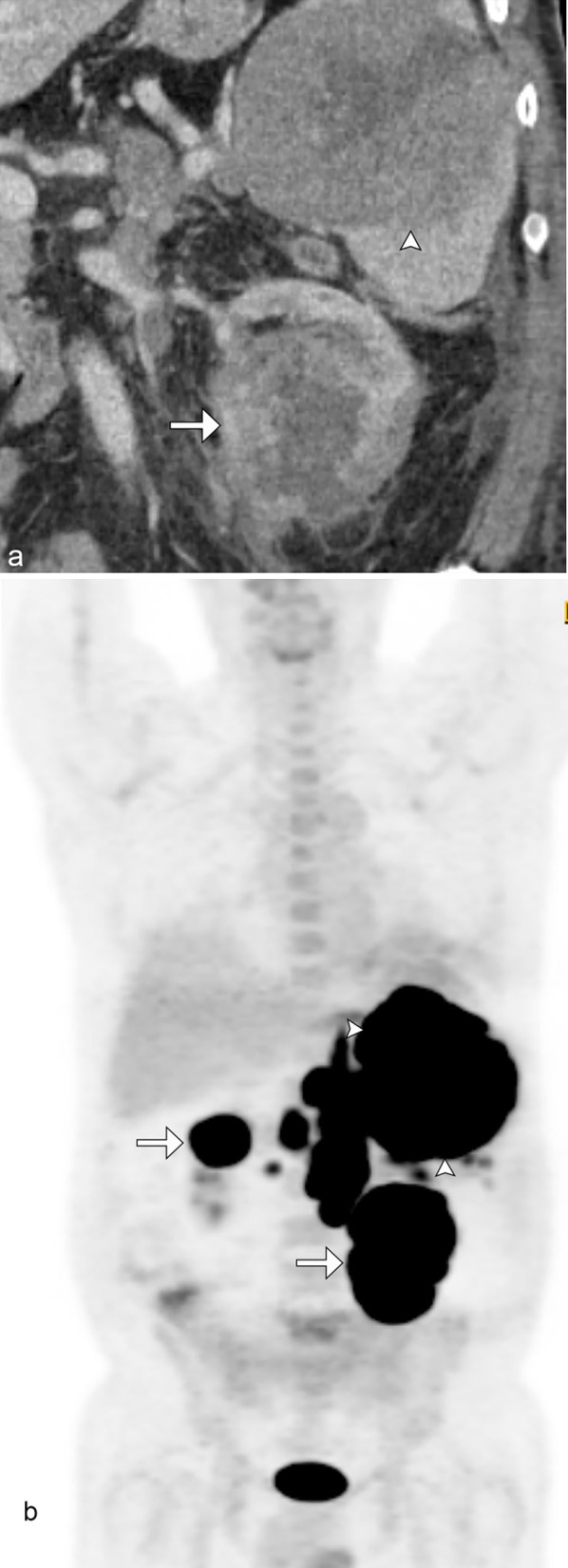
*Solitary mass of renal lymphoma.* Coronal CT post contrast image (**a**) demonstrates a solitary hypoenhancing left lower pole renal mass (arrow). Note the mass-like infiltration of the spleen (arrowhead) and retroperitoneal lymphadenopathy. PET/CT (**b**) shows the marked F18-FDG avid bilateral renal masses (arrows), retroperitoneal lymphadenopathy, and splenic mass (arrowheads), consistent with the diagnosis of large b-cell lymphoma

In approximately 10–25% of patients, renal lymphoma will present as a solitary mass [1, 4, 8]. When there is a lack of lymphomatous involvement elsewhere, a prospective diagnosis can be challenging (Fig. 2). Conversely, when retroperitoneal lymphadenopathy and splenic disease are present, these are helpful clues in diagnosing secondary renal lymphoma (Figs. 3 and 4).

Other differential diagnoses for a solitary renal lesion include solitary metastasis and other benign etiologies such as focal pyelonephritis, abscess, or infarct.

### Direct extension

**Fig. 5:**
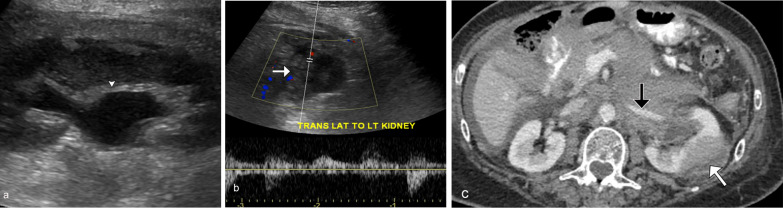
*Direct extension from retroperitoneal adenopathy of renal lymphoma.* Grayscale ultrasound image (**a**) demonstrates mild hydronephrosis (arrowhead) of the left kidney. Spectral Doppler ultrasound (**b**) demonstrates a hypoechoic mass (*white arrow*) arising from the lateral left kidney with arterial waveform. Axial contrast CT (**c**) confirms the left renal mass (*white arrow*) with additional extensive retroperitoneal and peritoneal infiltrative disease encasing the left renal hilum. Note the maintained flow and normal caliber left renal artery (*black arrow*) despite tumor burden, characteristic of lymphoma. This patient is known to have follicular lymphoma with high grade B-cell lymphoma transformation

Direct lymphomatous renal involvement from retroperitoneal lymphadenopathy is the second most commonly observed pattern of renal lymphoma, documented in approximately 25–30% of cases [5]. These patients usually have widespread disease with bulky tumors invading the perinephric space, displacing or invading the adjacent kidney. Resultant hydronephrosis from entrapment of ureters is common. However, vascular occlusion or thrombosis of renal arteries and veins is rare (Fig. 5).

In the absence of a known diagnosis, other retroperitoneal malignant etiologies such as sarcomas may be considered. Other benign etiologies mimicking this process include IGG-4-related disease and retroperitoneal fibrosis (RPF) [19].

### Diffuse renal infiltration or nephromegaly

**Fig. 6:**
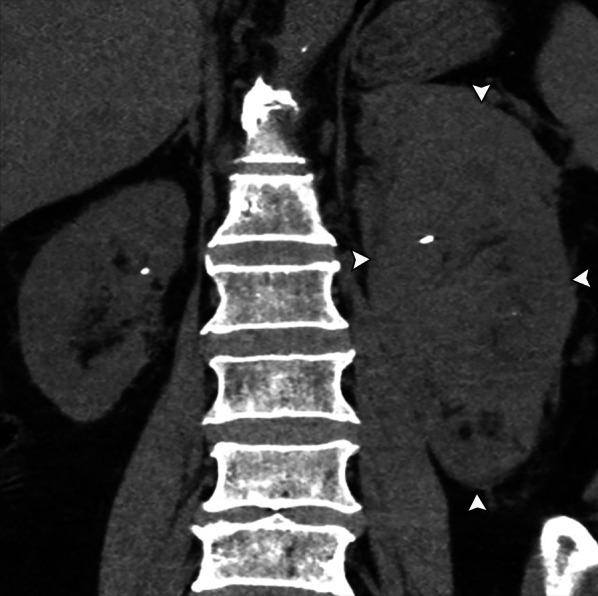
*Nephromegaly of renal lymphoma.* Coronal noncontrast CT demonstrates infiltrative renal lymphoma with a diffusely enlarged left kidney replaced by tumor (arrowheads). Note the encasement and deformity of the pelvocalyceal system by tumor. This was pathologically confirmed as marginal zone lymphoma

**Fig. 7:**
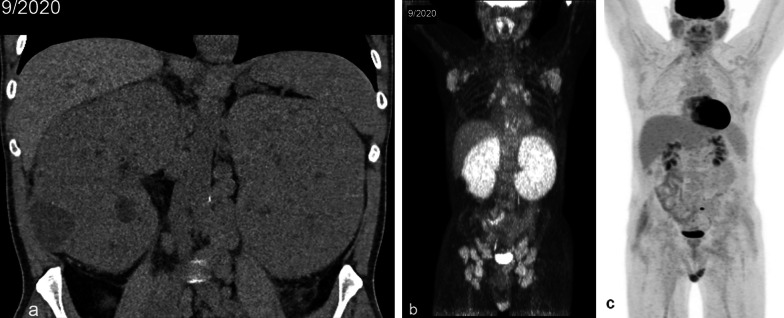
*Nephromegaly of renal lymphoma*. Coronal noncontrast CT (**a**) demonstrates symmetric marked enlargement of the bilateral kidneys from lymphomatous infiltration in this patient with mantle cell lymphoma. Concurrent PET/CT in 9/2020 (**b**) demonstrates marked F18-FDG avid bilaterally enlarged kidneys as well as extensive supradiaphragmatic and infradiaphragmatic lymphadenopathy. Follow-up F18-FDG PET/CT in 11/2020 (**c**) following chemotherapy shows complete metabolic and radiologic response with normalized renal size and activity

Renal lymphoma resulting in nephromegaly without distortion of the normal reniform shape has been reported in 20% of cases. Lymphomatous proliferation in the interstitium of the kidney results in nephromegaly. This may be disseminated or limited to the kidneys and may be unilateral or bilateral (Figs. 6 and 7). Patients may present with acute renal failure from the destruction of the normal renal architecture.

On CT, the kidney appears enlarged, often with heterogeneous enhancement, loss of normal corticomedullary differentiation, and possible infiltration of the renal sinus fat [4].

Differential considerations include urothelial carcinoma, medullary renal cell carcinoma, acute autoimmune nephritis, and severe pyelonephritis [20].

### Perinephric space disease

**Fig. 8:**
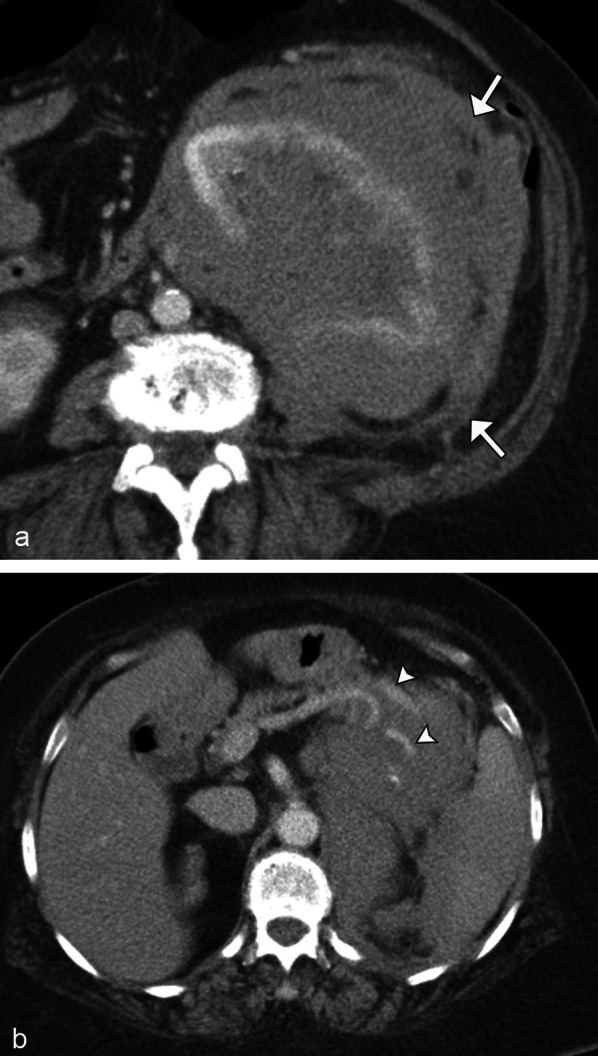
*Perinephric space infiltration of renal lymphoma*. Axial CT of the abdomen (**a**) demonstrates an infiltrative homogenous mass (arrows) in the left retroperitoneum enveloping the left kidney with preservation of the left renal parenchymal anatomy. Additional thickening of Gerota’s fascia and Zuckerkandl fascia as well as retroperitoneal tumor infiltration along the pancreatic tail and splenic hilum are present (**b**). Note the preservation of normal caliber splenic vein and artery (arrowheads). Due to the unusual tumor morphology, the left renal mass was biopsied and consistent with diffuse large B-cell lymphoma

Isolated perinephric lymphoma is unusual and reported in less than 10% of cases [6, 10]. The imaging findings include limited thickening of Gerota’s fascia, plaques and nodules within the perirenal space, or a rind of perinephric soft tissue thickening with invasion or compression of the normal renal parenchyma (Fig. 8) [4].

The differential diagnosis includes sarcoma, metastases to the perinephric space, and benign conditions such as pancreatitis, perinephric hematoma, RPF, amyloidosis, and extramedullary hematopoiesis (EMH).

### Renal sinus involvement

Lymphoma can preferentially affect the renal sinus, though this is a very rare phenomenon and the exact incidence is not well documented.

**Fig. 9:**
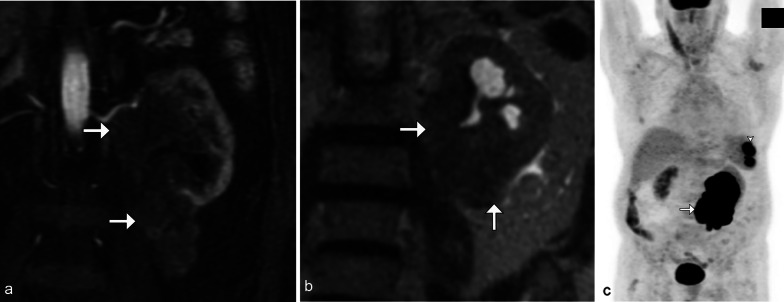
*Renal sinus involvement of renal lymphoma.* Coronal MRI post contrast (**a**) and T2 weighted (**b**) images demonstrate a T2 hypointense, hypoenhancing infiltrative left renal mass (arrows) invading the upper pole collecting system and lower kidney. Note the resultant mild hydronephrosis relative to tumor size. PET/CT (**c**) shows the marked F18-FDG avid left renal mass (arrow) as well as splenic masses (arrowhead). This was biopsy-proven high-grade B-cell lymphoma

On imaging, the renal sinus is replaced by a homogenous soft tissue mass, often resulting in mild hydronephrosis relative to the size of the tumor, due to its pliable nature. Vascular encasement or displacement is commonly seen (Fig. 9). On US, it may be difficult to differentiate the renal sinus tumor from heterogenous renal sinus fat due to its poorly defined margins [4, 21].

## Benign mimics

### Pyelonephritis and renal abscesses

**Fig. 10:**
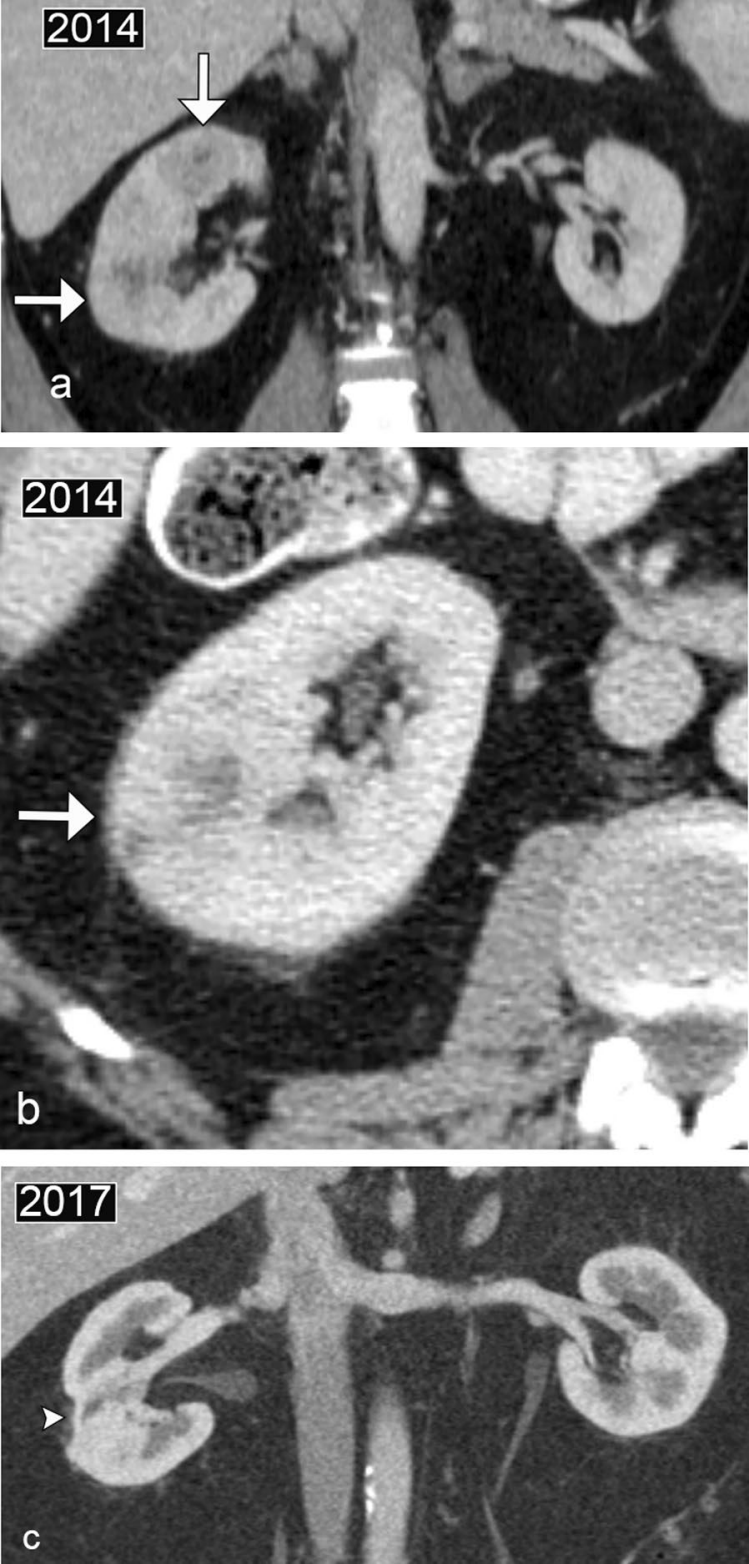
*Pyelonephritis and renal abscesses.* Coronal (**a**) and axial (**b**) contrast CT images in 2014 of the abdomen demonstrate a delayed right nephrogram with cortical heterogeneity. Suggestion of mass-like areas in the right kidney with perinephric stranding (arrows) identified, mimicking the multiple masses of renal lymphoma. This was presumed to be related to pyelonephritis with renal abscesses based on clinical presentation. Follow-up coronal contrast CT in 2017 (**c**) demonstrates focal parenchymal scarring in the region of the right lower pole mass as a result of prior infection (arrowhead)

Focal pyelonephritis and abscesses may mimic renal masses and renal lymphoma. Clinical information is essential in making the diagnosis, including symptoms of dysuria, hematuria, flank pain, and a urinalysis positive for infection. On CT, a striated nephrogram or areas of patchy hypoenhancement can be seen in pyelonephritis, with a predilection for the upper pole. On ultrasound, renal abscesses appear as complex collections, with posterior through transmission, and lack internal vascularity [22]. On contrast-enhanced imaging, abscesses may demonstrate rim enhancement. Follow-up imaging often demonstrates focal parenchymal scarring as a result of prior inflammation/infection (Fig. 10).

### IGG4 related renal disease

**Fig. 11:**
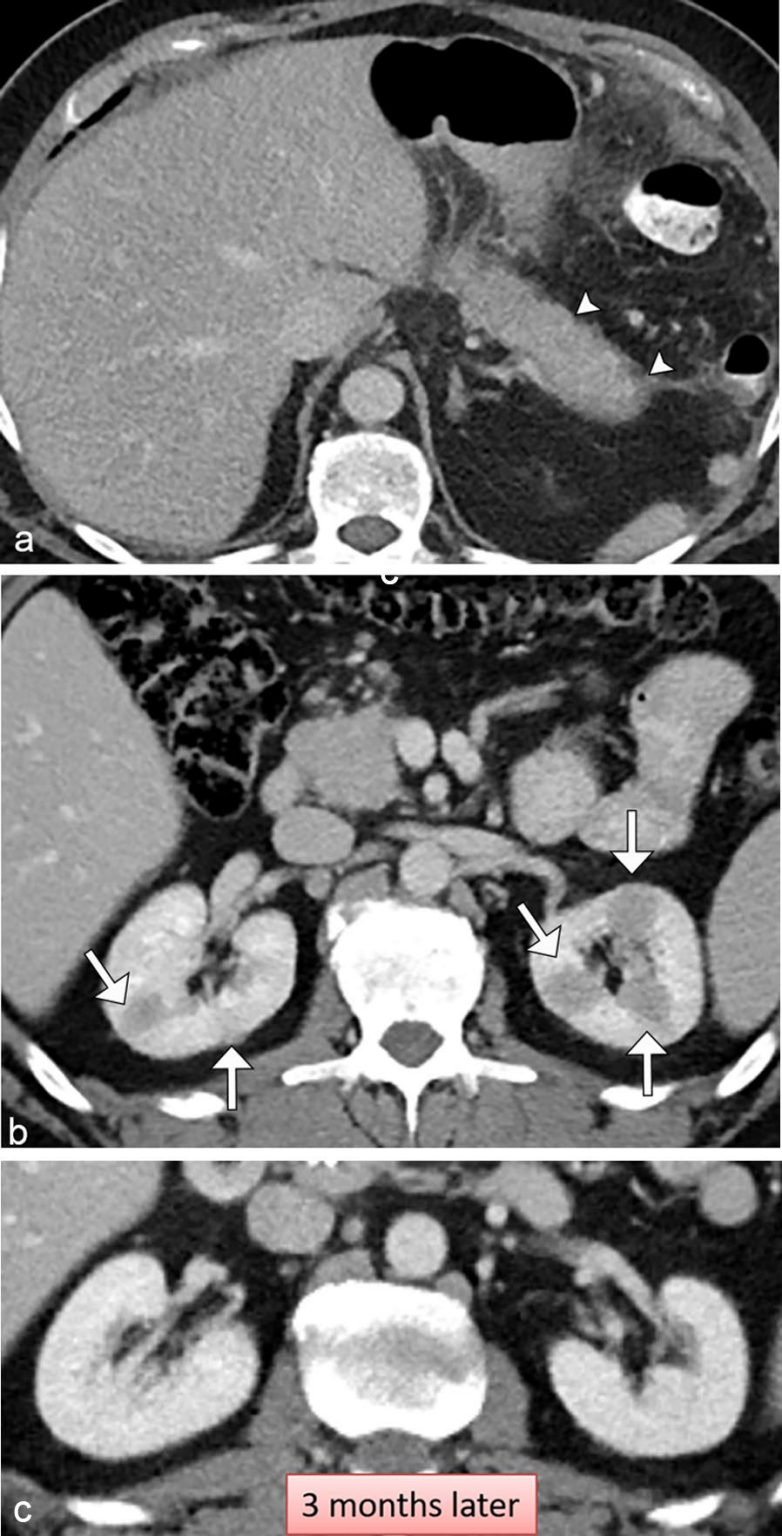
*IGG4-related renal disease*. Axial contrast CT images demonstrate (**a**) a sausage-shaped pancreas with loss of normal pancreatic lobulations and presence of a hypoattenuating surrounding halo/capsule (arrowheads) consistent with IGG4-related autoimmune pancreatitis. (**b**) Bilateral hypoattenuating renal masses (arrows) were present compatible with IGG4-related renal disease in light of the pancreatic findings, mimicking the multiple masses of lymphoma. Axial contrast CT 3 months later (**c**) post-treatment with steroids demonstrates resolution of the renal masses confirming the initial diagnosis

IGG4-related disease is characterized by fibroinflammatory lesions rich in IGG4 positive plasma cells, and often but not always elevated serum IGG4 concentrations. Five patterns of disease have been described in renal involvement, with bilateral round or wedge-shaped peripheral cortical lesions being the most common pattern (Fig. 11) [23]. Patients usually improve after corticosteroid treatment. On MRI, IGG4 lesions demonstrate both T1 and T2 hypointense signal with mild enhancement on post-contrast T1 weighted images [24].

### Extramedullary hematopoiesis

**Fig. 12:**
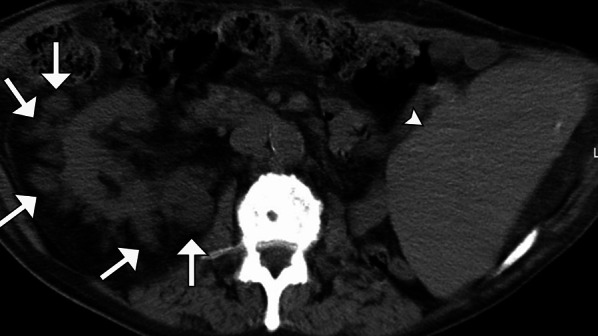
*Renal extramedullary hematopoiesis.* Axial noncontrast CT in an anemic patient demonstrates splenomegaly (arrowhead) and multiple right renal masses (arrows). The constellation of findings is suspicious for underlying lymphoma. A biopsy of one of the renal masses shows hematopoietic tissue consistent with extramedullary hematopoiesis

EMH is the proliferation of hematopoietic tissue in response to profound chronic anemia in sites other than the medullary cavity [25]. Renal EMH is a rare phenomenon and only a few cases have been reported. Lesions are typically homogenous, mimicking renal cell carcinoma, and can be multifocal (Fig. 12), involve the renal sinus, and may be F18-FDG avid on PET/CT [26]. In patients with myelofibrosis, a diagnostic clue is the coincide advanced marrow fibrosis as a result of reticulin deposition, which manifests as increased marrow density. In patients with beta-thalassemia, there may be signs of iron overload from repeated transfusions. Biopsy is usually necessary for definitive diagnosis, with pathology demonstrating hematopoietic rests.

### Retroperitoneal fibrosis

**Fig. 13:**
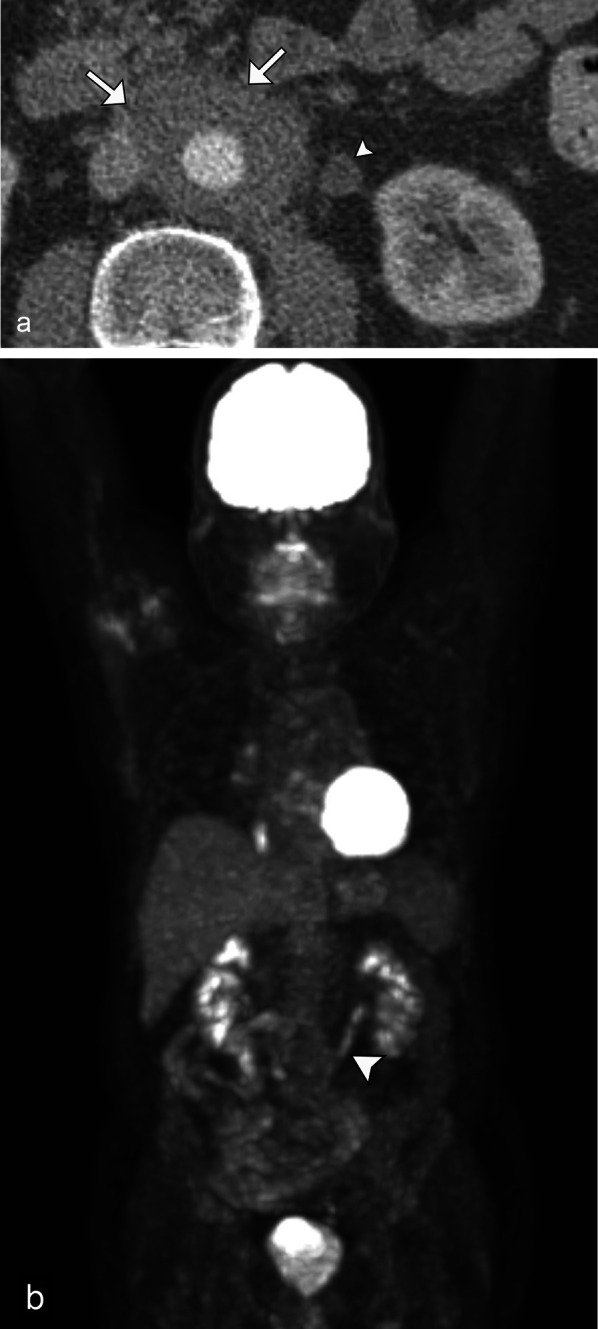
*Retroperitoneal fibrosis.* Axial contrast CT (**a**) of the abdomen demonstrates retroperitoneal mass-like soft thickening enveloping the aorta without lifting it anteriorly (arrows), and a dilated left ureter (arrowhead). This mimics retroperitoneal lymphomatous disease, although lymphoma tends to lift/displace the aorta/IVC. F18-FGD PET/CT MIP (**b**) demonstrates a medialized left ureter (arrow). Note the lack of FDG avidity in the expected region of the retroperitoneal mass. Findings are consistent with retroperitoneal fibrosis

RPF is characterized by the proliferation of fibroinflammatory tissue occurring as a primary disease (75% of cases) or secondary to other processes (25% of cases). Primary RPF can be related to systemic autoimmune or inflammatory diseases such as IGG4-related disease. Secondary RPF may occur as a complication of therapy or neoplasm [27]. On CT, RPF is usually homogenous and irregular periaortic soft tissue. It often envelops the aorta, inferior vena cava, and ureters without displacing these structures anteriorly from the spine, as would be seen in lymphoma. Medialization of the ureters with hydroureteronephrosis may result from the surrounding fibrosis (Fig. 13) [27]. On MRI, RPF typically appears as a homogeneously low signal mass on T1 weighted imaging, with a variable signal on T2 depending on the disease stage. Increased T2 signal can be seen in the early fibroinflammatory process whereas decreased T2 signal and delayed enhancement are seen in chronic fibrosis [27].

### Erdheim–Chester disease

**Fig. 14:**
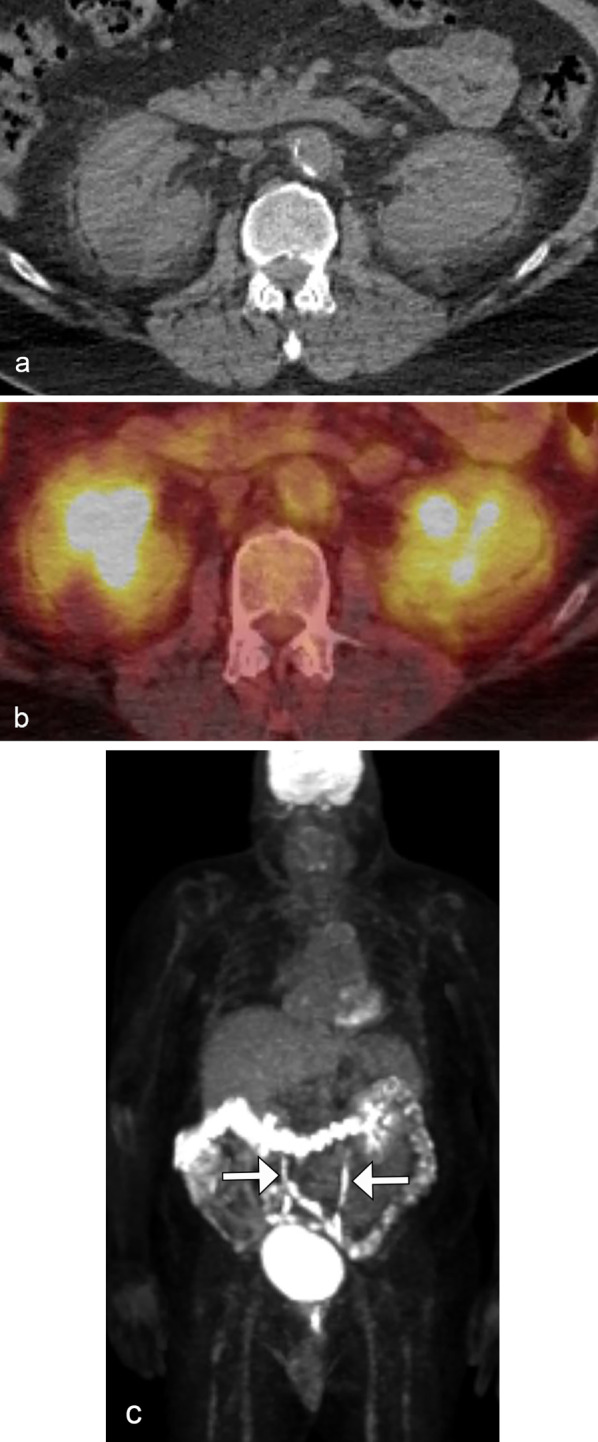
*Erdheim–Chester disease.* Axial noncontrast CT (**a**) demonstrates bilateral perinephric soft tissue stranding/infiltration mimicking the perinephric infiltrative appearance of lymphoma. F18-FDG PET/CT images (**b**, **c**) demonstrate the low-grade metabolic activity associated with soft tissue stranding. Note the normal course of the bilateral ureters (arrows). The patient was also found to have thoracic periaortic soft tissue thickening and bilateral metadiaphyseal sclerosis which are additional features of Erdheim–Chester disease

Erdheim–Chester disease (ECD) is a non-Langerhans cell histiocytosis characterized by multiorgan xanthomatous infiltration, involving the skeleton in 96% of reported cases [28]. In the abdomen, ECD can present as retroperitoneal infiltration in one-third of patients and appears similar to RPF. The kidneys may demonstrate enhancing perirenal soft tissue (Fig. 14), with or without obstructive uropathy [29].

## Malignant mimics

### Urothelial carcinoma

**Fig. 15:**
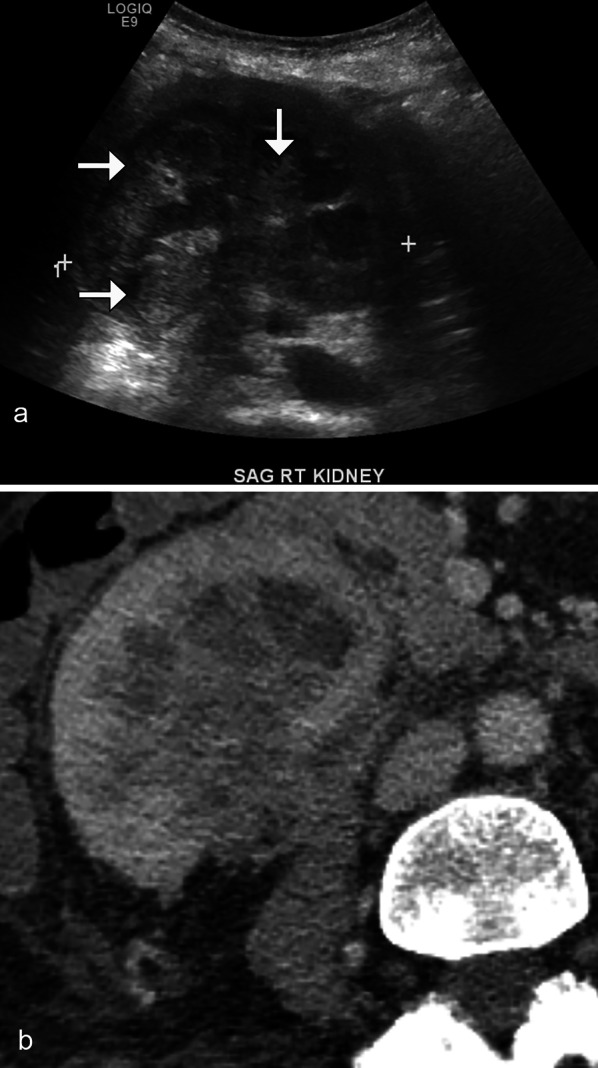
*Urothelial carcinoma.* Sagittal grayscale ultrasound (**a**) demonstrates an enlarged right kidney with relative preservation of the reniform shape. Note the diffuse infiltration of the pelvocalyceal system (arrows). Contrast-enhanced axial CT (**b**) shows the diffuse enlargement of the right kidney with infiltrative tissue centered in the collecting system; subsequent biopsy confirmed urothelial carcinoma, mimicking the diffuse infiltration/nephromegaly pattern of renal lymphoma

Urothelial carcinoma (UC) typically involves the bladder (90% of cases). Approximately 8% of UC cases have been reported in the kidney, with preferential involvement of the extrarenal part of the renal pelvis than the infundibulocalyceal portion. Hematuria is the most common symptom, reported in 95% of patients. CT urography is critical in tumor assessment with renal UC typically appearing as an irregular filling defect or area of wall thickening, often infiltrating the renal parenchyma (Fig. 15). There may be resultant hydronephrosis depending on the location of the tumor [30]. On MRI, the tumor may appear isointense to slightly hypointense on T1 weighted imaging, isointense to mildly hyperintense on T2 weighted imaging, with mild, heterogenous enhancement post-contrast [31].

### Renal cell carcinoma

**Fig. 16:**
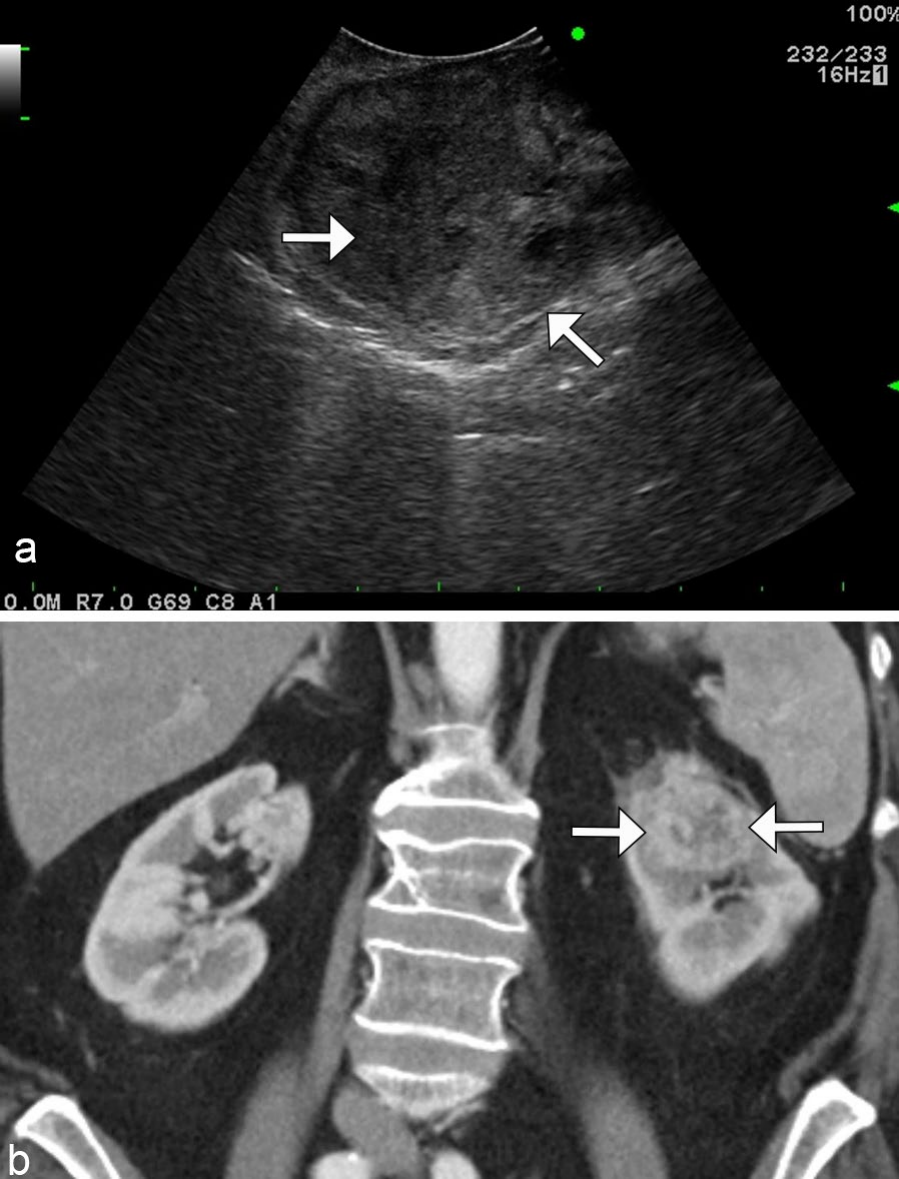
*Clear cell renal cell carcinoma.* Sagittal grayscale ultrasound (**a**) demonstrates a heterogeneous right upper pole renal mass (arrows). Coronal contrast CT (**b**) demonstrates enhancement of the renal lesion (arrows). Biopsy confirmed renal cell carcinoma. Note the increased vascularity and heterogeneity relative to what would typically be seen in a solitary renal lymphomatous lesion

RCC is the most common malignant epithelial tumor of the kidney, accounting for 90% of all solid renal tumors in adults. Clear cell RCC is the most common subtype of RCC, occurring in up to 65–80% of cases [32]. These are typically hypervascular tumors and may appear heterogeneous due to necrosis, cystic degeneration, or hemorrhage. They often enhance heterogeneously during the arterial phase, and more avidly than other RCC subtypes (Fig. 16). Up to 60% of clear cell RCCs demonstrate the presence of intralesional microscopic fat, which may appear as a drop in MR signal intensity on out-of-phase imaging [33]. Clear cell RCC tends to invade vessels, most often the renal vein and inferior vena cava. Compared with papillary and chromophobe RCCs, clear cell carcinomas are more likely to be encountered at an advanced stage and with metastases [32].

**Fig. 17:**
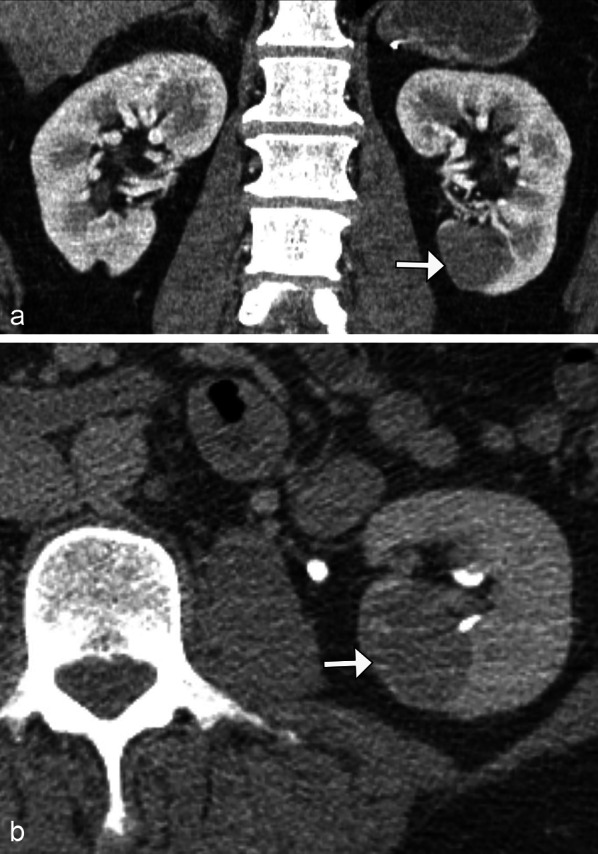
*Papillary renal cell carcinoma.* Coronal (**a**) and axial (**b**) contrast CT images demonstrate a hypoenhancing left lower pole renal mass (arrows), which was biopsy confirmed as papillary renal cell carcinoma mimicking the solitary hypoenhancing mass of lymphoma

Papillary RCC is the second most common subtype of RCC, accounting for 10–15% of cases. Papillary RCC typically presents as a well-circumscribed mass, peripherally located, and may have internal hemorrhage or necrosis when larger than 4 cm in size [33]. Typically, papillary carcinomas appear hypointense on T2 weighted imaging and are hypoenhancing, similar to the solitary mass appearance of renal lymphoma (Fig. 17). A biopsy may be necessary to differentiate these two entities.

### Imaged-guided percutaneous biopsy

Renal lymphoma has a variable imaging appearance and can mimic a broad range of benign and malignant entities. When there is a lack of other diagnostic clues such as a known history of lymphoma, concurrent retroperitoneal nodal or splenic involvement, the prospective diagnosis can be challenging. Some indications for renal biopsy include non-characterizable lesion by imaging, suspected renal lymphoma, confirmation of metastasis in the setting of other known malignancy, before ablation therapy, etc. Image-guided percutaneous renal biopsy can be performed under ultrasound or CT guidance.

Ultrasound-guided renal biopsy has the benefit of real-time imaging without exposing the patient to radiation. This technique is typically employed for nontargeted renal biopsy or when the renal mass is large enough to be targeted with ultrasound. Typically, the patient is placed in a lateral decubitus or prone position, allowing for a short trajectory percutaneously. Contrast-enhanced ultrasound can be employed to improve needle visualization and differentiate solid tissue from necrosis [34]. However its use in real-time renal biopsy has not been reported.

Conversely, CT-guided renal biopsy has the benefit of increased spatial resolution and contrast, especially when the lesion is small, deep, or superior location making it difficult to be visualized with ultrasound. CT-guided biopsy can also be performed with intravenous iodinated contrast, allowing better visualization of the lesion and surrounding critical structures such as vasculature. CT-guided biopsy allows for various approaches including anterior, posterior, or lateral trajectory [35].

Both modalities allow immediate post-procedural assessment for complications such as hematoma, vascular or adjacent organ injury. Percutaneous renal biopsy is increasingly performed and considered safe, effective, and minimally invasive, with a very low risk of seeding along the biopsy tract [35].

## Conclusion

The kidneys are the most common genitourinary site of involvement of extranodal lymphoma. Renal lymphoma has a wide spectrum of imaging appearances, often mimicking other benign and malignant conditions, presenting a diagnostic dilemma for radiologists. The typical patterns of renal involvement include solitary or multiple lesions, retroperitoneal tumors directly invading the kidneys, unilateral or bilateral renal enlargement, perirenal soft tissue infiltration, and renal sinus involvement. Radiologists should be familiar with both typical and atypical manifestations of renal lymphoma. Appropriate recommendations for additional imaging or image-guided biopsy when indicated may obviate unnecessary surgery.

## Data Availability

Not applicable.

## Abbreviations

CEUS: Contrast-enhanced ultrasound
CT: Computed tomography
ECD: Erdheim–Chester disease
EMH: Extramedullary hematopoiesis
MRI: Magnetic resonance imaging
PET: Positron emission tomography
PET/CT: Positron emission tomography/computed tomography
RCC: Renal cell carcinoma
RPF: Retroperitoneal fibrosis
UC: Urothelial carcinoma
